# Macrophages/Microglia in the Glioblastoma Tumor Microenvironment

**DOI:** 10.3390/ijms22115775

**Published:** 2021-05-28

**Authors:** Jun Ma, Clark C. Chen, Ming Li

**Affiliations:** Department of Neurosurgery, University of Minnesota, Minneapolis, MN 55455, USA; jma603@umn.edu

**Keywords:** glioblastoma, tumor microenvironment, glioblastoma-associated macrophages/microglia, macrophage, microglia

## Abstract

The complex interaction between glioblastoma and its microenvironment has been recognized for decades. Among various immune profiles, the major population is tumor-associated macrophage, with microglia as its localized homolog. The present definition of such myeloid cells is based on a series of cell markers. These good sentinel cells experience significant changes, facilitating glioblastoma development and protecting it from therapeutic treatments. Huge, complicated mechanisms are involved during the overall processes. A lot of effort has been dedicated to crack the mysterious codes in macrophage/microglia recruiting, activating, reprogramming, and functioning. We have made our path. With more and more key factors identified, a lot of new therapeutic methods could be explored to break the ominous loop, to enhance tumor sensitivity to treatments, and to improve the prognosis of glioblastoma patients. However, it might be a synergistic system rather than a series of clear, stepwise events. There are still significant challenges before the light of truth can shine onto the field. Here, we summarize recent advances in this field, reviewing the path we have been on and where we are now.

## 1. Glioblastoma Associated Macrophages/Microglia

The dynamic, complex interaction between Glioblastoma (GBM) and its microenvironment has been recognized, which endows the tumor cells with the potent ability to escape immune surveillance, resist various interventions, and survive. Immune profiling of the GBM microenvironment reveals diversified profiles, including myeloid cells, glioblastoma-associated macrophages and microglia (GAMs), T-lymphocytes, NK cells, granulocytes, dendritic cells, etc., although both the transcriptional and the morphological characteristics of these cells may have changed significantly. Pimenta analyzed the profiles of primarily human GBM and reported three distinct immune profiles: cases with a minor fraction of leucocytes, tumors with a predominance of GAMs and neutrophils, and cases with mixed infiltration by GAMs, neutrophils, and T-lymphocytes. Untreated GBM patients with mixed myeloid and lymphoid immune infiltrates showed a significantly shorter overall patient survival [1]. Fu et al. confirmed that GAMs, as the dominant infiltrating immunocytes, have great inter- and intra-tumoral heterogeneity. GAMs, increased exhausted T cells, infiltrating Tregs, and nonfunctional NK cells contribute to local immune suppressive characteristics. Recurrent GBM share similar immune signatures with the initial GBM, except the proportion of GAMs decreases [2].

### 1.1. Concepts about GAMs

In GBM, GAMs represent the major population with up to one half of the cells of the tumor mass. Bone-marrow-derived macrophages and monocytes cells (BDMC) accounted for 85% of total GAMs, while resident microglia accounted for the remaining 15% [3]. The present definition of such cells is largely based on the cell markers. In the past, the resting macrophage is considered as M0, while M1 and M2 represent polarized macrophages. Previously, it has been considered that the M1 components mediate pro-inflammatory functions, while the M2 compartments mediate anti-inflammatory function [4]. However, with further understanding of the population, it is highly likely that GAMs are composed of heterogeneous subpopulations [5,6]. Here, we refer to these related populations as pro-tumoral macrophage and anti-tumoral macrophage to avoid confusion by the arbitrary nomination.

Due to the dynamic switching of the cells on both the genetic and phenotypic levels, as well as the homologous relationship between macrophage and microglia, it might be more accurate to interpret any concepts by the identification markers used. The combination of multiple markers is a good choice before the highly specific markers are identified. Macrophages are frequently expressed CD45 and c-MAF (musculoaponeurotic fibrosarcoma). CCL3 is an anti-tumoral macrophage marker, while CD68 and CD163 mark pro-tumoral macrophages [7]. The mRNA expression profile of GAMs displays an upregulation of factors that are considered as anti-tumoral macrophages (e.g., CCL2, CCL3L3, CCL4, PTGS2) and pro-tumoral macrophage polarization markers (e.g., MRC1, LGMN, CD163, IL10, MSR1). It has also been reported that human GBM contain mixed polarized GAMs. A higher amount of CD68-, CD163-, and CD206-positive GAMs in the vital tumor core was associated with beneficial patient survival [8]. Some adopt CCR2 to code macrophages and CX3CR1 for microglia [9]. It is demonstrated that CX3CR1^Lo^CCR2^Hi^ monocytes are recruited to the GBM, where they transit to CX3CR1^Hi^CCR2^Lo^ macrophages and CX3CR1^Hi^CCR2^-^ microglia-like cells [3].

### 1.2. Macrophage versus Microglia in GBM Microenvironment

The resident microglia could not be avoided whenever talking about GAMs, and in fact, macrophage and microglia are hard to separate from each other exclusively. Both of their transcriptional and morphological patterns are unique to infiltrating and resident cells [9]. Single-cell RNA sequencing and CITE-seq revealed a large and diverse myeloid compartment in GBM. Microglia- and monocyte-derived GAMs are self-renewing populations that compete for space and could be depleted via a CSF1R blockade. Microglia-derived GAMs are predominant in newly diagnosed tumors but are outnumbered by monocyte-derived GAMs following recurrence, especially in hypoxic environments [10]. The total number of microglia cells does not vary with the grade of malignancy, but macrophage-like cells prevail in high-grade gliomas. Microglia favor tumor progression, but many aspects suggest that the phagocytosing function is maintained. Moreover, microglia correlates with poorer survival in GBM when considering CD163+ cells, whereas it does not change prognosis in isocitrate dehydrogenase (IDH)-mutant low grade gliomas [11]. One mechanism is that GBM-initiating cells induce the mTOR-dependent regulation of STAT3 and NF-κB activity in the microglia, which promotes an immunosuppressive microglial phenotype. This hinders effector T-cell infiltration, proliferation, and immune reactivity, thereby contributing to tumor immune evasion and promoting tumor growth. But this phenomenon is not observed in BDMCs [12]. Another study showed that the subpopulation consisting of CD45^high^-expressing cells (macrophages) is activated by the tumor primarily with suppressive as well as pro-inflammatory characteristics, whereas the CD11b^+^CD45^low^ cells (microglia) are almost unaffected [13]. The predominance of pro-tumoral macrophages leads to the suppression of local and systemic immunity in high-grade gliomas [7].

It is believed that there are different preferential distributions and functions of GAMs in different locations, as well as in different GBM subtypes. In an immunohistochemical analysis of a panel of immune biomarkers within the GBM microenvironment, the CD163^+^ pro-tumoral macrophages appeared to be the most common cell type in both the peritumoral area (PTA) and the tumor core (TC) [14]. Similarly, another study proved that in central tumor areas and around vessels in the infiltration zone there were more CD68+ GAMs in slow-growing tumors. Central tumor areas contained more GAMs compared with the infiltration zone. GAMs are present in high numbers in most regions of the tumor, whereas there are few in necrotic areas. Furthermore, slow-growing GBMs seem to contain a GAM-population different to their fast-growing counterparts [15]. There are distinct regional ontogeny and activations of GAMs. Core GAMs evolve towards a pro-inflammatory state and a subpopulation of cells are identified with a strong, opposing correlation with Programmed cell Death-1 (PD-1) signaling, which may correlate to their response to PD-1 inhibition. Meanwhile, peripheral GAMs evolve towards anti-inflammatory phenotypes and contain a population of cells strongly associated with NF-kB signaling. The result advocated the need for a multitargeted approach to GBM therapeutics [16]. BDMCs are recruited to the tumor early during GBM initiation and localized preferentially to perivascular areas. Resident microglia are localized mainly to peritumoral regions [3]. A co-expression of glioma biomarkers (GFAP, IDH1R132H, and PDGFRA) and macrophage biomarkers (CD68 and CD14) is also noticed on some cells, defined as GAM-GBM hybrid cells. These hybrids gather in the margin of GBM and are enriched with glioma invasion-associated genes. They believe that the hybrids undergo reprogramming of the GAM so that they possess higher invasiveness than their parental cells [17]. GAMs are also the main immune cells of hypoxic pseudopalisades in GBM, which is linked to tumor malignancy and aggressiveness. The cells migrate to necrotic areas to clear the resulting components of the prothrombotic milieu, suggesting that the scavenging features of GAMs at the pseudopalisades serve as an essential counterpart to glioma cell invasion [18].

GAMs are reported to be more abundant in mesenchymal GBM and gliomas with many gemistocytic cells. The amount of CD204+ GAMs (pro-tumoral macrophages) especially increases with the malignancy grade and may contribute to a pro-tumorigenic microenvironment. The CD204+ GAMs co-expressed proteins related to tumor aggressiveness including matrix metallopeptidase-14 (MMP14) and hypoxia-inducible factor-1α (HIF-1α). In grade III-IV, high CD204 expression is associated with shorter survival, while high IBA-1 intensity correlates with a longer survival. In grade IV, CD204 shows an independent prognostic value when adjusting for clinical data and the methylation status of O6-methylguanine-DNA methyl transferase (MGMT) [19]. There are fewer GAMs in IDH-mutant GBM, but they are more pro-inflammatory, while anti-inflammatory macrophages that upregulate genes such as FCER1G and TYROBP predominate in IDH-wild type GBM. These more pro-inflammatory GAMs in IDH-mutant GBM are associated with longer overall survival independent of IDH status [20].

There are a few more studies with gender considered. Lisi et al. looked into the markers of iNOS, CD163, and ARG-1 for microglial polarization, with the consideration of gender. CD163 expression is higher within the tumor than in surrounding periphery in both male and female patients; while iNOS is higher within the tumor in males, no significant difference is found for ARG-1 [21]. Turaga et al. reported an interesting result. They found that junctional adhesion molecule A (JAM-A)-deficient female mice succumb to GBM more quickly compared with WT females and JAM-A-deficient and male WT mice. It has been revealed that the microglia in tumors of females with JAM-A-deficient microglia are more activated, and RNA-sequencing identified elevated expressions of Fizz1 and Ifi202b specifically in JAM-A-deficient female microglia. This highlights an emerging role for sex differences in the GBM microenvironment and suggests that sex differences extend beyond tumor cell-intrinsic differences [22].

## 2. The Synergistic System

To get a simple glimpse of the GBM-Macrophage/Microglia integration, the present model describes how glioma tumor cells recruit GAMs into the tumor microenvironment (TME), and after infiltrating the cells, are activated, polarized, and reprogrammed to fulfill their duties in tumor development and invasion. Unfortunately, there are no ideal stepwise processes that could be intervened at any specific point. These are much more complicated processes in which the tumor cells and the environmental cells are working synergistically, with multiple signaling pathways involved.

### 2.1. GBM on GAMs

Generally, various signaling pathways are involved in GAMs remodeling to make them the battle companion of GBM. Glioma stem cells (GSCs) secrete the Wnt-induced signaling protein 1 (WISP1) to promote the survival of both GSCs and GAMs, thus facilitating a pro-tumor microenvironment [22]. Glioma initiates CD133(+) cells and the macrophages/microglia cointeraction activates the expression of B7-H4 (B7x/B7S1), a B7 family member, via IL6 and IL10 in both tumor cells and microenvironment-supporting cells. IL6-activated STAT3 binds to the promoter of B7-H4 gene and enhances B7-H4 expression. CD133(+) cells also mediate immunosuppression through B7-H4 expression on macrophages/microglia by silencing the B7-H4 expression, leading to an increased microenvironment T-cell function and tumor regression [23]. The loss of CD47 function upregulates the tumor-associated extracellular matrix protein tenascin C (TNC) expression in tumor cells via a Notch pathway-mediated mechanism. TNC stimulates the release of proinflammatory factors, including TNFα via a Toll-like receptor 4 and a STAT3-dependent mechanism in human macrophage cells [24]. Limiting monocyte infiltration via genetic CCL2 reduction prolongs the survival of tumor-bearing mice [3]. EGFR and epidermal growth factor variant III (EGFRvIII) cooperate to induce macrophage infiltration via upregulation of the chemokine CCL2, and KRAS was a critical signaling intermediate for the EGFR- and EGFRvIII-induced expressions of CCL2 in glioma cells [25]. CCL8 is a GAM-associated factor to mediate the invasion and stemness of GBM [26]. Vascular adhesion protein (VAP-1) abundance is closely linked to alternative pro-tumoral activation during glioma progression [27]. CSF2 (macrophage colony stimulating factor) is overexpressed in a subset of mesenchymal GBM in association with high immune gene expression. Tumor-derived CSF2 attracts, supports the survival of, and induces the pro-tumorigenic polarization of microglia and macrophages [28].

Taking the concept of stepwise functioning of GAMs, the GBM influence runs through all the processes. Both tumor-derived and host-derived osteopontin (OPN) are critical for glioma development. It’s concluded that OPN is an important chemokine for recruiting macrophages to GBM, mediating crosstalk between tumor cells and the innate immune system [29]. SET domain bifurcated 1 (SETDB1) is upregulated in GBM and promotes AKT/mTOR-dependent CSF-1 induction and secretion, which leads to macrophage recruitment in the tumor, resulting in tumor growth. Thus it is relative to poor progression [30]. There is an ERK1/2-dependent regulation of the production of the macrophage chemoattractant CCL2. GBM with high levels of phosphorylated ERK1/2 demonstrate increased infiltration of GAMs with a non-inflammatory M2 polarization [31]. Coagulation factor X (FX) is highly expressed in and positively correlated with GAM density in human GBM. FX is secreted in the tumor microenvironment and increases the phosphorylation and activation of the extracellular signal-related kinase (ERK)1/2 and AKT in macrophages, exhibiting a potent chemotactic capacity to recruit macrophages and promoting macrophages toward pro-tumoral subtype polarization, thus accelerating GBM growth. CASC2c interacts with and reciprocally represses miR-338-3p. They both bind to FX and inhibit its expression and secretion. Thus, CASC2c represses pro-tumoral macrophage polarization [32]. CD200 is a type I transmembrane glycoprotein and can interact with its receptor CD200R, which plays an inhibitory role in the activation of microglia of the central nervous system. The downregulation of CD200 expression in CD200-rich glioma cells could foster the formation of an activated microglia-associated tumor microenvironment, leading to glioma progression [33]. P-selectin mediates microglia-enhanced GBM proliferation and invasion by altering the microglia/macrophages’ activation state. The pharmacological and molecular inhibition of P-selectin leads to reduced tumor growth and increased survival in GBM mouse models [34]. Protein disulfide-isomerase A3 (PDIA3) expression plays a role in the GBM-mediated pro-tumoral activation of microglia. Reduced PDIA3 expression/activity in GBM cells significantly limits the microglia pro-tumor polarization towards the pro-tumoral phenotype and the production of pro-inflammatory factors [35]. Monocytes are the precursors to macrophages. GSCs-derived exosomes (GDEs) have a predilection for monocytes, traverse the monocyte cytoplasm, cause a reorganization of the actin cytoskeleton, and skew monocytes toward the immune suppressive pro-tumoral phenotype, including programmed PD-L1 expression [36]. ARS2 (arsenite-resistance protein 2) is a zinc finger protein essential for early mammalian development. ARS2 plays critical roles in GSC maintenance and pro-tumoral macrophage-like GAM polarization [37]. GAMs are critical mediators in the PD-1/PD-L1 axis. miR-106b-5p expression is upregulated in glioma-infiltrating macrophages, where miR-106b-5p regulates pro-tumoral polarization of GAMs and enhances the growth of glioma-infiltrating macrophages. IRF1 was identified as a target of miR-106b-5p. miR-106b-5p inhibits IRF1 expression by targeting IRF1/IFN-β pathway to promote the pro-tumoral polarization of macrophages [38]. Angiopoietin-2 (Ang-2) overexpression compromised the benefit from anti-VEGF therapy in a preclinical GBM model. In animal models, targeting Ang-2/VEGF with A2V antibodies reprograms the pro-tumoral M2 macrophages toward the anti-tumoral macrophage phenotype. Therefore, A2V may prolong survival in mice with GBM by reprogramming the tumor immune microenvironment and delaying tumor growth [39]. Osteopontin and lactadherin are proteins that cooperatively activate amoeboid transformation, phagocytosis, and the motility of primary microglia cultures via integrins and FAK-Akt (focal adhesion kinase-Akt) signaling. Osteopontin/secreted phosphoprotein 1 (Spp1) produced by non-transformed cells acts as a proinflammatory factor, inducing inflammatory signaling and anti-tumoral macrophage genes and counteracting the action of lactadherin [40]. T-excreted branched-chain ketoacids (BCKAs) can be taken up and re-animated to branched-chain amino acids (BCAAs) by tumor-associated macrophages. Exposure to BCKAs reduced the phagocytic activity of macrophages [41]. The disruption of the SIRPα-CD47 signaling axis is efficacious against various brain tumors, including GBM, primarily by inducing tumor phagocytosis, and even in the absence of phagocytizing macrophages (Ccr2 RFP/RFP), microglia are effector cells of tumor cell phagocytosis in response to anti-CD47 blockade [9].

### 2.2. GAMs on GBM

GAMs also have their role on GBM. They trigger a Proneural-to-Mesenchymal Transition (PMT) in GSCs via small extracellular vesicles (sEV). sEVs from monocyte-derived macrophages transfer miR-27a-3p, miR-22-3p, and miR-221-3p to GSCs, and these miRNAs promote several mesenchymal phenotypes in proneural GSCs by simultaneously targeting CHD7 [42]. The concept of MARCO^high^ (a collagenous structure) GAMs has been reported, which are cells originating from peripheral blood whose master regulators and target genes are often associated with genomic aberrations in neurofibromin 1 (NF1) and the phosphoinositide 3-kinases/mammalian target of rapamycin/Akt pathway (PI3K-mTOR-AKT)-related genes. They induce a phenotypic shift towards the mesenchymal cellular state of GSCs, promoting both invasive and proliferative activities, as well as therapeutic resistance to irradiation. The potential association with tumor-induced polarization states and immunosuppressive environments is also suggested [43]. The RNA regulator HuR is a key modulator of pro-glioma responses by reprogramming microglia/macrophages through the molecular regulation of chemokines, cytokines, and other factors. It stimulates anti-tumor immunity and attenuates glioma growth [44]. The Na(+)/H(+) exchanger isoform 1 (NHE1) function plays an important role in glioma-microglia interactions, enhancing glioma proliferation and invasion by stimulating the microglial release of soluble factors [45]. CD11b+/CD163+ GAMs secrete abundant pleiotrophin (PTN) to stimulate GSCs through its receptor PTPRZ1, promoting GBM malignant growth [46]. GAMs of human GBM specimens and of a syngeneic glioma model express CCR2 to varying extents. Lacking CCR2 solely on tumor microenvironmental cells leads to enhanced tumor progression, whereby high numbers of GAMs infiltrate gliomas independently of the CCR2/CCL2 signal [47]. Cat Eye Syndrome Critical Region Protein 1 (CECR1) is a potent regulator of GAMs polarization and is consistently, highly expressed by M2-type GAMs, particularly in high-grade glioma. Paracrine effects induced by CECR1 in pro-tumoral macrophage-like GAMs activate MAPK signaling and stimulate the proliferation and migration of glioma cells [48].

### 2.3. Crosstalk between GBM and GAMs

The ultimate picture is still viewing GBM & GAMs as an integrated interaction system. CXCL16 is released by tumor cells. CXCL16/CXCR6 signaling promotes the modulation of GAMs toward an anti-inflammatory/pro-tumor phenotype, and also restrains microglia polarization toward an inflammatory phenotype upon LPS and IFNγ stimulation. It has also been demonstrated that CXCL16/CXCR6 signaling acts directly on the primary GBM cells, promoting tumor cell growth, migration, and invasion [49]. Chen et al. established a symbiotic glioma–macrophage interplay in PTEN-deficient GBM, proving that PTEN deficiency activates YAP1, which directly upregulates lysyl oxidase (LOX) expression. The secreted LOX functions as a potent macrophage chemoattractant via activation of the β1 integrin-PYK2 pathway in macrophages. The infiltrating macrophages secrete SPP1, which sustains glioma cell survival and stimulates angiogenesis [50]. The miR-340-5p-macrophage feedback loop is suggested. It has been found that miR-340-5p levels are correlated with the density of GAMs and M2-polarized GAMs in GBM. Downregulation of miR-340-5p promotes GAMs recruitment through directly targeting POSTN, which recruits GAMs through integrin αvβ3, and also promotes pro-tumoral GAMs polarization through directly targeting LTBP-1. Furthermore, pro-tumoral GAMs, which promote tumorigenesis, inhibit miR-340-5p expression in GBM cells by the upregulation of TGFβ-1, which increases HMGA-2 expression in GBM [51]. GBM-derived exosomes (GBex) reprogram anti-tumoral and pro-tumoral macrophages, converting anti-tumoral macrophage into GAMs and augmenting pro-tumoral macrophage functions. In turn, these GBex-reprogrammed GAMs, produce Arginase-1+ exosomes, which further disseminate immunosuppressive and tumor-growth promoting proteins, promoting tumor cell migration and proliferation [4]. The levels of a pro-tumoral, macrophage-specific secreted cytokine, TGF-β1, are elevated in the presence of GSCs. The co-culture of CSCs and macrophages results in enhanced expression of the pro-tumoral macrophage markers in macrophages that are previously polarized to the anti-tumoral macrophage phenotype, thus the co-culture results in a bidirectional signaling that alters the phenotypes of both cell types [52]. Autotaxin (also termed ATX or Enpp2) is an enzyme that synthetizes Lysophosphatidic acid (LPA) which is a lysophospholipid that acts as a bioactive signaling molecule. Microglial cells express high levels of Autotaxin, suggesting a microglia-GBM interaction through the LPA pathway with relevant implications for tumor progression [53].

Neuropilin 1 (NRP1) expression is correlated with poor prognosis and glioma grade, and associates with the mesenchymal GBM subtype. In human GBM, NRP1 expression is highly correlated with the markers of monocytes/macrophages, as well as genes that contribute to the pro-tumorigenic phenotype of these cells [54]. The LGALS protein family is a large class of sugar-binding proteins. Hu et al. reported LGALS3 as an independent poor prognostic marker in diffusely infiltrating gliomas and it was also positively correlated with immune cell infiltration, particularly CD163+ tumor-associated macrophages in the TCGA dataset, Rembrandt dataset, and their SYSUCC cohort [55]. Huang et al. screened for transcriptional expression levels and methylation data of an OS (overall survival)-correlated gene EFEMP2 (EGF containing fibulin-like extracellular matrix protein 2). It was observed that the resting macrophage, as a feature of malignancy of GBM, revealed a distinct assembly in glioma with high levels of EFEMP2, supporting EFEMP2′s role as a marker of assembly of M0 macrophage and more malignant phenotypes of glioma [56]. The expression levels of ADAM10 and ADAM17 are significantly correlated with an anti-tumoral, macrophage-like phenotype and are positively associated to patient survival. Whilst ADAM8 mRNA expression was equally correlated with anti-tumoral and pro-tumoral, macrophage-like markers, genes for MMP9 and MMP14 are significantly associated with a pro-tumoral, macrophage-like phenotype and are further associated with an impaired prognosis in the GBM patient cohort [57]. Cerebrospinal fluid (CSF) interleukin-6 (IL-6) levels, one of the pleiotropic cytokines that has received attention as a critical factor implicated in the invasion and the angiogenesis of various cancers, are associated with the infiltration rate of GAMs in GBM, indicating that the concentration of CSF IL-6 may be a useful prognostic biomarker for the GBM patients [58].

### 2.4. Other Mechanisms

Other ingredients in the GBM microenvironment cannot be neglected. The recruited NK cells mediate a major part of the phytosomal curcumin (CCP)-evoked elimination of GBM and GSCs and of the stabilization of the GAMs in the anti-tumoral, macrophage-like state. The brain-released chemokine CCL2 activates the secretion of IL12 by the peripheral anti-tumoral macrophage, which activates NK cells and causes the destruction of the GBM cells and GSCs [59]. Tumor cells and GAMs have a role in inducing CD4+Foxp3- type 1 regulatory T (Tr1) cells in GBM patients [60]. Kynurenine produced by GBM cells activates the aryl hydrocarbon receptor (AHR) in GAMs to modulate their function and T cell immunity. AHR promotes CCR2 expression and also drives the expression of KLF4 while suppressing NF-κB activation in GAMs and drives the expression of the ectonucleotidase CD39 in GAMs, which promotes CD8+ T cell dysfunction by producing adenosine in cooperation with CD73. High AHR expression was associated with poor prognosis [61].

Busch et al. also reported consistent expression of immunoglobulins in GAMs and circulating monocytes from all of their patient GBM samples. The immunoglobulin repertoires of circulating monocytes and GAMs are generally more restricted compared to B cells. These immunoglobulin expressions in the macrophage populations negatively correlate with the tumor’s volume [62]. Wang et al. mentioned hypoxia, which enhanced the interaction between macrophages and GBM cells by upregulating CCL4 and CCR5 expression, while the CCL4–CCR5 axis plays a role in the macrophage-promoted invasion of GBM [63]. Dusoswa et al. considered the GBM glycocalyx as a tumor-intrinsic immune suppressor. They detected increased expression of both tumor-associated truncated O-linked glycans and the receptor, macrophage galactose-type lectin (MGL), on CD163+ GAMs in GBM patient-derived tumor tissues. In an immunocompetent orthotopic glioma mouse model overexpressing truncated O-linked glycans (MGL ligands), high-dimensional mass cytometry revealed a wide heterogeneity of infiltrating myeloid cells with increased infiltration of PD-L1+ GAMs as well as distant alterations in the bone marrow. They concluded that these tumor-associated glycans trigger inhibitory signaling in GAMs through glycan-binding receptors [64]. Xu et al. looked into α-l-fucosidase 1 (FUCA1), a lysosomal enzyme that catalyses the hydrolytic cleavage of the terminal fucose residue, has been reported to be involved in tumorigenesis. Lower levels of tumor-infiltrating macrophages, including CD68+ (~30%), F4/80+ (~50%), and CD11c+ macrophages (~50%), were identified in FUCA1-downregulated glioma tissues, and CCL2/CCL5 neutralizing Abs blocked this effect. This suggested that the downregulation of α-l-fucosidase 1 suppresses glioma progression by enhancing autophagy and inhibiting macrophage infiltration [65].

## 3. Therapeutic Benefits

Theoretically, all above mentioned research results could offer more perspectives for GBM treatment. In addition, there are some studies focusing on specific therapies.

### 3.1. Radiation

Anti-tumoral macrophages seem to be more sensitive to ionizing radiation than pro-tumoral macrophages, both in normoxia and in hypoxia. Pro-tumoral macrophages are more radioresistant than resting and anti-tumoral macrophages. Leblond et al. proposed that X-ray radiotherapy could contribute to the increased density in the pro-tumoral macrophage in GBM [66]. In a study of the combined treatment of high-dose radiation and immunotherapy, radiation treatment was found to trigger macrophage repolarization, increasing the anti-tumoral/pro-tumoral macrophage ratio. This combined treatment appears to be highly effective with a 75% complete pathologic response and dramatically improved survival outcomes. Furthermore, CD8+ T-cells and macrophages are necessary for the full effect of the combined therapy, with T lymphocytes playing a role early on and macrophages mediating a later phase of the treatment [67]. The increased infiltration of anti-tumoral macrophages was associated with a poor radiation response for IDH-wild-type GBM, and the anti-tumoral macrophages correlated with WHO grades and were robustly predicted for the survival performance for GBM patients [68].

### 3.2. Anti-Angiogenic Treatment

Macrophages/microglia represented the main source of alternative proangiogenic factors, and tumor-infiltrating myeloid cells might play a crucial role in the limited efficacy of anti-angiogenic therapy, bypassing VEGF-mediated pathways through the expression of alternative proangiogenic factors [69]. Various studies support the use of anti-angiogenics in combination with immunotherapy, considering that the pro-inflammatory repolarization of perivascular and perinecrotic GAMs is paramount for overcoming treatment resistance [70]. Bevacizumab is a typical anti-angiogenic drug. Its resistance is driven by reduced MIF at the tumor edge, causing the proliferative expansion of pro-tumoral macrophages [69]. It has also been found that suppression of CCL2 can play an important role in increasing the efficacy of anti-angiogenic treatment in GBM by inhibiting the recruitment of CCL2-dependent macrophages [71]. Kim et al. developed a mathematical and computational model that involves reaction–diffusion equations for the important components in the tumor–microglia interaction, including the densities of the tumor and microglial cells, and the concentrations of growth factors and other signaling molecules, serving as a 3-D model of the mechanical and biochemical interactions between a GBM and the tumor microenvironment. With this model they demonstrated that microglia can stimulate tumor cell invasion by secreting the growth factor TGF-β [72]. Su et al. reported that MRX-2843, a MerTK inhibitior, has a therapeutic benefit via promoting GAMs polarization away from immunosuppressive conditions, inhibiting neoangiogenesis in the GBM microenvironment, and inducing tumor cell death [73]. Li et al. reported ADAM8, a metalloprotease-disintegrin strongly expressed in tumor cells and associated immune cells of GBMs, is related to angiogenesis and correlates with poor clinical prognosis. The angiogenic potential of ADAM8 in GBM cells and in primary macrophages is mediated by the regulation of osteopontin. ADAM8 could be a tractable target to modulate angiogenesis in GBM [74]. Zhu et al. found that surgical debulking with anti-CD47 blocking immunotherapy causes a decrease in angiogenic proteins besides enhanced inflammatory response and prolonged survival in animals [75].

### 3.3. PD-L1 Signaling Pathway

In de Groot et al.’s study, recurrent GBM tumors contained few T cells that demonstrate a paucity of immune activation markers, however, the tumor microenvironment is markedly enriched for CD68+ macrophages. Pembrolizumab anti- PD-1 monotherapy alone cannot induce an effective immunologic response in most GBM patients, probably owing to a scarcity of T cells within the tumor microenvironment and a CD68+ macrophage preponderance [76]. In the late temozolomide (TMZ) treatment or relapse stage, anti-PD-L1 antibody treatment significantly reduced the infiltration of CD163-positive macrophages into tumors, while a combined PD-L1 antibody and IPI-549 (a Selective PI3Kγ Inhibitor) therapy remarkably inhibited tumor growth [77]. Rapamycin and hydroxychloroquine (RQ) treatment was found to decrease the macrophages polarization of M2, increase the phagocytic ability, and increase the accumulation of lipid droplets. This treatment enhanced the intra-tumoral anti-tumoral/pro-tumoral ratio and the CD8/CD4 ratio. The combination of RQ and anti-PD1 treatments is synergistic in action [78]. Saha D et al. tested a triple combination of anti-CTLA-4, anti-PD-1, and G47Δ-mIL12 (oncolytic herpes simplex viruses armed with angiostatin and IL-12) in mouse GBM models. The treatment was associated with macrophage influx and anti-tumoral, macrophage-like polarization, along with increased T effector to T regulatory cell ratios, and cured most mice with glioma. Immune cell depletion studies demonstrated that CD4^+^ and CD8^+^ T cells as well as macrophages are required for synergistic curative activity [79]. Reactive oxygen species (ROS) modulator 1 (Romo1) is a mitochondrial membrane protein that is essential for the regulation of mitochondrial ROS production and redox sensing. Romo1 is highly expressed in macrophages. In the GBM mouse model, the overexpression of Romo1 in bone marrow cells was found to significantly inhibit the immune response within tumor microenvironment, and the overexpression of Romo1 resulted in the pro-tumoral macrophage polarization of bone marrow derived macrophages (BMDMs) through the mTORC1 signaling pathway. The inhibition of Romo1 combining with anti-PD-1 immunotherapy significantly improves the survival outcome of GBM in mouse models [80].

### 3.4. PI3Kγ Signaling Pathway

We recently reported that GAMs secrete IL11 to activate STAT3-MYC signaling in GBM cells, which induces stem cell states that confer enhanced tumorigenicity and resistance to TMZ. PI3Kγ is abundantly expressed in myeloid cells but not cancer cells and promotes myeloid cell trafficking during inflammation and cancer. We found that the inhibition or inactivation of PI3Kγ reduces microglia recruitment and IL11 secretion, which in turn promotes exceptional TMZ response in the mouse GBM models [81]. Quail et al. demonstrated that in recurrent GBM, PI3K pathway activity was elevated, driven by macrophage-derived IGF-1 and tumor cell IGF-1 receptors (IGF-1R). Meanwhile, macrophages accumulate with GBM progression and can be targeted via the inhibition of the colony-stimulating factor-1 receptor (CSF-1R). Therefore, they combine IGF-1R or PI3K blockade with CSF-1R inhibition in recurrent tumors, which significantly prolonged overall survival [82].

### 3.5. Miscellaneous Medical Treatment Targeting GBM-GAM Interaction

It has been found in bone marrow-derived macrophages exposed to glioma conditioned media (GCM), oxaliplatin (OXA)-reduced levels of phosphorylated STAT3 and decreased expressions of ARG1 independent of apoptosis induction [83]. Endothelial cells (ECs) are one of the major sources for IL-6 expression in the GBM microenvironment. CSF-1 and angiocrine IL-6 induce robust ARG1 expression and macrophage alternative activation through the peroxisome proliferator-activated and receptor-γ-dependent transcriptional activation of HIF-2α. The EC-specific knockout of IL-6 inhibits macrophage alternative activation and improves survival in the GBM-bearing mice [84]. Liaw et al. introduced a dendrimer-triptolide conjugate that specifically targets GAMs in GBM from systemic administration and exhibits triggered release under intracellular and intratumoral conditions. This targeted delivery improves phenotype switching of GAMs from pro-tumor towards anti-tumor expression [85]. ONC201/TIC10 is an anti-cancer molecule that antagonizes the dopamine receptor D2 and affects mitochondria integrity in tumor cells. ONC201 affects macrophage immunometabolism and leads to a pro-inflammatory tumor environment. Macrophages responded to ONC201 with a severe loss of mitochondria integrity, a switch to glycolytic ATP production, alterations in glutamate transport, and a shift towards a pro-inflammatory profile [86]. Zhang et al. used a targeted nanocarrier that can deliver in vitro-transcribed mRNA encoding anti-tumoral macrophage-polarizing transcription factors to reprogram GAMs without causing systemic toxicity. They demonstrated that infusions of nanoparticles formulated with mRNAs encoding interferon regulatory factor 5, in combination with its activating kinase IKK*β* ≤, reverse the immunosuppressive, tumor-supporting state of GAMs and reprogram them to a phenotype that induces anti-tumor immunity and promotes tumor regression [87]. There is a study exploring the possibility of exploiting the cross-talks between the GBM cells and GAMs for modulation of the GBM microenvironment through the use of Nano-DOX, a drug composite based on nanodiamonds bearing doxorubicin. Nano-DOX-damaged GC exhibited an enhanced ability to attract both GAMs and Nano-DOX-loaded GAMs and reprogrammed the GAMs from a pro-GBM phenotype to an anti-GBM phenotype that suppressed GC growth [88]. FTY720 is a potent immunosuppressant. FTY720 could inhibit the growth, migration, and invasion of glioma by targeting GAMs to impede their effect on glioma cells, while also potentially blocking the chemoattraction of GAMs by inhibiting the MAPK-mediated secretion of IL-6 through the increased internalization of CXCR4. Microglia and macrophages are polarized from pro-glioma to an anti-tumor phenotype [89]. Zhang et al. showed that GAMs display a continuum of different polarization states between anti-tumorigenic and pro-tumorigenic macrophage phenotypes. A lower anti-tumoral/pro-tumoral ratio correlates with a worse prognosis. Anti-CD47 treatment shifts the phenotype of macrophages toward the anti-tumoral subtype, leading to enhanced tumor cell phagocytosis by both anti-tumoral and pro-tumoral macrophage subtypes with a higher phagocytosis rate caused by anti-tumoral macrophages [90]. Chlorogenic acid (5-caffeoylquinic acid, CHA), a phenolic compound with low molecular weight, has an anti-tumor effect in multiple malignant tumors. CHA serves as a potential therapeutic approach to reduce glioma growth through promoting anti-tumoral macrophages and inhibiting pro-tumoral macrophages [91]. Ocoxin^®®^ oral solution (OOS) has anti-inflammatory and antioxidant properties, as well as anti-tumor properties in several neoplasia without known side effects. OOS has a direct effect on macrophage polarization, inhibiting the pro-tumoral features of macrophages and slowing down their tumor growth in certain GBM [92]. GAMs secrete exosomes that are enriched with miR-21. Treatment with pacritinib and the combination of pacritinib and TMZ appears to significantly reduce the tumorigenesis of GBM/GAMs PDX mice as well as overcome TMZ resistance and pro-tumoral polarization of GAMs. Either pacritinib alone or in combination with TMZ could suppress GBM tumorigenesis via modulating STAT3/miR-21/PDCD4 signaling [93]. The enhancement of zeste homolog 2 (EZH2) inhibition leads to a significant reduction of TGFβ1-3 and IL10 and elevation of IL1β and IL6, which participates in GBM-induced immune deficient. EZH2 suppression in GBM cells can remodel microglia immune functions, resulted in significant increase of anti-tumoral macrophage markers (TNFα and iNOS) and decrease of a pool of pro-tumoral macrophage markers, ameliorated phagocytic capacities of microglia, and declined microglia proliferation (with addition of TGFβ2 antibodies) to co-incubated GBM cells with EZH2 inhibition. There is also an enhanced engulfment microglia through the activation of iNOS [94].

### 3.6. Novel Drug Delivery Methods and Platforms

Specific targeting GAMs is an ideal therapeutic strategy for GBM intervention. For instance, A dendrimer-rapamycin conjugate (D-Rapa) that specifically targets GAMs in GBM from systemic administration is presented. D-Rapa localizes specifically within GAMs, acting as depots to release rapamycin into the tumor microenvironment. In vitro, it improves the suppression of pro-tumor expression in activated GAMs and the anti-proliferative properties of rapamycin in glioma cells [95]. In another study, a novel dendrimer conjugated to the translocator protein (18 kDa) (TSPO) ligand 5,7-dimethylpyrazolo [1,5-α]pyrimidin-3-ylacetamide (DPA) is presented. These dendrimers can achieve GAMs-specific targeting in GBM and can be further modified to target specific intracellular compartments for organelle-specific drug delivery [96]. Targeted delivery with CHA-encapsulated mannosylated liposomes enhances the immunotherapeutic efficacy of CHA by inducing a shift from the pro-tumoral to the anti-tumoral macrophage phenotype effectively [97]. Local oncolytic virotherapy Delta24-RGD may promote a prolonged shift in the pro-tumoral macrophages toward anti-tumoral macrophages in human GBM, inducing a pro-inflammatory and potentially tumor-detrimental microenvironment [98]. Some developed a virus-mimicking, membrane-coated nucleic acid nanogel Vir-Gel embedded with a therapeutic miRNA, miR155, which can reprogram microglia and macrophages from a pro-invasive macrophage phenotype to an anti-tumoral macrophage phenotype. The virus-mimicking nucleic acid nanogel provides a general and convenient platform that prolongs the circulation lifetime of miR155 and endows it with an active tumor-targeting capability and excellent tumor inhibition efficacy [99]. The constructed Schizophyllan (SPG) nanoparticles entrapping CpG ODN 1826 are capable of crossing the blood–brain barrier. In rat models, the intracellular oxidative burst and cytokine levels pre- and post-incubation with nanoparticles exhibit a marked elevation in the expression of intracellular ROS, IFN-γ, and IL-1β post treatment. This indicates towards the potentiality of repolarizing the pro-tumoral macrophage to an anti-tumoral phase by inducing high levels of oxidative burst and inflammatory cytokines [100]. Liposomal TriCurin (TrLp) administration into mice yields a stable plasma concentration of 210 nM C for 60 min, which is able to cause the repolarization of pro-tumoral, macrophage-like GAMs to the tumoricidal, anti-tumoral, macrophage-like phenotype and the intra-GBM recruitment of activated natural killer cells. These tumoricidal immune cells are associated with the concomitant suppression of the tumor-load, and the apoptosis of GBM and GSCs [101].

Gardell et al. genetically engineered human monocyte-derived macrophages to secrete a bispecific T cell engager (BiTE) specific to the mutated EGFRvIII expressed by some GBM tumors. They found that such human genetically engineered macrophages (GEMs) can locally and constitutively express one or more therapeutic proteins, which may help recruit T cells and transform the immunosuppressive tumor microenvironment to better support anti-tumor immunity [102].

### 3.7. Combined Therapy

Given that resected tumors have a relatively large population of pro-tumoral macrophages in GBM at the baseline, which are increased in response to radiotherapy, Almahariq et al. tested the CSF-1R inhibitor BLZ-945, which reduces pro-tumoral macrophage polarization and improves the response to radiotherapy [103]. Ene et al. found that anti-PD-L1 immunotherapy enhances a radiation-induced abscopal response via canonical T cell activation and direct macrophage activation in GBM [104]. Wu et al. reported that MerTK/CD68+ macrophages increase in recurrent tumors, and suggested that MerTK inhibition combined with fractionated external beam radiotherapy (XRT) has a therapeutic effect in a subset of GBM [105]. Similarly, Su et al. reported that MRX-2843, a MerTK inhibitor, alleviates immunosuppression as well as inhibits neoangiogenesis in the glioma microenvironment. They observed decreased vascular formation and numbers of immunosuppressive (CD206+) GAMs following MRX-2843 treatment, inducing tumor cell death [73]. Zhu et al. revealed an increase in recruitment of cells positive for CD68 positive macrophages to the surgical site. They also demonstrated an increase in pro-inflammatory cytokines, such as CXCL10, and a decrease in angiogenic proteins in debulking plus anti-CD47 vs. non-debulking plus IgG tumors. Thus, they suggested that surgical resection combined with anti-CD47 blocking immunotherapy promotes an inflammatory response and prolongs survival [75]. Herting et al. used dexamethasone, which turned out to block IL-1 production in both bone marrow-derived and brain resident macrophage populations following stimulation with lipopolysaccharide and interferon gamma. They suggested the IL-1 signaling as a therapeutic target for the management of GBM-associated cerebral edema [106].

## 4. The Next Step

Defining cells is the first priority. There are controversies regarding a functional phenotype of macrophages and microglia due to a lack of consistent markers. Walentynowicz et al.’s analyses of marker genes in GAMs from different experimental models and clinical samples revealed only a small set of common genes, which reflects the variegated responses in clinical and experimental settings. *Tgm2* and *Gpnmb* were the only two genes common in the analyzed data sets. There is a great need for definitive elucidation of a functional state of GAMs [107]. Given such tremendous diversity, appropriate markers will help clarifying what exactly we are looking at.

The research models, platforms, and methods are improving. An et al. described a quantitative cell-based assay to assess macrophage recruitment by the conditioned medium from the tumor cells. They used human macrophage cell line MV-4-11 to study macrophage attraction by the conditioned medium from GBM, showing high reproducibility and low variability [108]. Coniglio et al. introduced a protocol to model GAM interaction using in vitro culture assays. Multiple pharmacological inhibitors (JNJ-28312141, PLX3397, Gefitinib, and Semapimod) have been identified to block macrophage/microglia-stimulated GBM invasion using these assays [109]. Lately, methods for generating and biobanking patient-derived GBM organoids (GBOs) that recapitulate the histological features, cellular diversity, gene expression, and mutational profiles of their corresponding parental tumors have been explored. GBOs maintain key features of GBM and can be rapidly deployed to investigate patient-specific treatment strategies. [110,111] In vivo, Hamilton L et al. described a live imaging assay to study glioma–microglia interactions in the zebrafish brain. They believe that this model will be an important tool for drug screening and development [112]. Foray et al. confirmed the efficiency of [18F] FET-PET for monitoring the TMZ-treatment response and demonstrated that in vivo TSPO-PET performed with [18F] DPA-714 can be used to identify specific reactive areas of myeloid cell infiltration in the TME [113]. Kim et al. developed a mathematical and computational model that involves reaction–diffusion equations for the important components in the tumor–microglia interaction, including the densities of tumors and microglial cells, and the concentrations of growth factors and other signaling molecules, serving as a 3-D models of the mechanical and biochemical interactions between a GBM and the tumor microenvironment. With this model, they demonstrated that microglia can stimulate tumor cell invasion by secreting TGF-β [72].

Noninvasive research methods critical for clinical studies. Zhou et al. investigated the MR Imaging characteristics associated with GAMs. It was concluded that radiologic necrosis, lack of a cystic component, and extensive peritumoral edema are more frequent in the alternative lengthening of telomeres (ALT)-/macrophages low-concentration (ALT-/M+) tumors. Furthermore, the cystic imaging feature is additive to the tumor subtype, and the MGMT status is used to predict improved patient survival [114]. Chen et al. used high-resolution open-skull two-photon microscopy to investigate the phenotypical and functional characteristics of GAMs. They demonstrated that BMDMs in GAMs are smaller, minimally branched cells that are highly migratory compared with microglia which are larger, highly branched stationary cells, and that two populations of monocytic macrophages were observed that differed in terms of CX3CR1 expression and migratory capacity. They also demonstrated the efficacy of anti-VEGFA blockades for prohibiting GAMs infiltration [115]. Karimian-Jazi et al. dissected the single steps of nanoparticle (NP) uptake by blood-borne monocytes that give rise to GAMs by magnetic resonance imaging and multiphoton microscopy (MR-MPM), allowing for innate immune cell tracking by MRI and multiphoton microscopy in the same animal to longitudinally investigate innate immune cell dynamics in the TME [116].

With improved research methods and better understanding of the mysterious GBM–GAMs world, we are heading towards that light.

## Data Availability

Not applicable.
